# Serum and urinary carnosinase-1 correlate with kidney function and inflammation

**DOI:** 10.1007/s00726-022-03206-9

**Published:** 2022-11-01

**Authors:** Shiqi Zhang, Di Cui, Mingna Tang, Guang Yang, Benito Yard, Huaqing Hu, Yonggui Wu, Qiu Zhang

**Affiliations:** 1 Department of Endocrinology, The First Affiliated Hospital of Anhui Medical University, Hefei, 230022 China; 2 Vth Department of Medicine (Nephrology/Endocrinology/Rheumatology), University Medical Center Mannheim, University of Heidelberg, 68167 Mannheim, Germany; 3 Health Management Center, The First Affiliated Hospital of Anhui Medical University, Hefei, 230022 China; 4 Department of Nephrology, The First Affiliated Hospital of Anhui Medical University, Hefei, 230022 China

**Keywords:** Diabetic nephropathy, Carnosinase, *CNDP1* gene, Inflammation, Methylglyoxal

## Abstract

The carnosinase dipeptidase 1 (*CNDP1*) gene has been reported as a susceptibility locus for the development of diabetic kidney disease (DKD). While the (CTG)_5_ allele affords protection in the Caucasian population, we have previously shown that this allele is less frequently present in the Chinese population and therefore a protective role for the (CTG)_5_ allele is difficult to demonstrate. In the present study, we sought to assess if carnosinase-1 (CN-1) concentrations in serum and/or urine are associated with progression of DKD and to what extent CN-1 influences diabetes-associated inflammation. From a total of 622 individuals that enrolled in our study, 247 patients had type 2 diabetes without DKD, 165 patients had DKD and 210 subjects served as healthy controls. Uni- and multivariate regression analyses were performed to identify potential factors predicting urinary albumin creatinine ratio (UACR), estimated glomerular filtration rate (eGFR) and CN-1 concentration in serum and urine. The results indicated that serum CN-1 indeed correlated with eGFR (*p* = 0.001). In addition, urinary CN-1 associated with eGFR and tubular injury indicator: urinary cystatin C (Cys-C) and urinary retinol-binding protein (RBP). Interestingly, serum CN-1 also positively correlated with inflammatory indicators: neutrophils and lymphocytes. With regard to this, a STZ injected C57BL/6 mice model with surgically made skin wound was established for the generation of skin inflammation. This animal model further proved that the expression of CN-1 in liver and kidney increased remarkably in diabetic mice with skin wound as compared to those without. In conclusion, serum and urinary CN-1 significantly related to the surrogates of impaired renal function in diabetic patients; besides, CN-1 expression might also be associated with the process of inflammation.

**Supplementary Information:**

The online version contains supplementary material available at 10.1007/s00726-022-03206-9.

## Introduction

Many genetic loci, including the carnosinase dipeptidase 1 (*CNDP1*) gene (Ahluwalia et al. 2011; Chakkera et al. 2011; Freedman et al. 2007a; Janssen et al. 2005; Mooyaart et al. 2011), have been reported to play a role in susceptibility to develop diabetic kidney disease (DKD). The *CNDP1* gene is located on chromosome 18q22.3 and contains a number of intronic and exonic polymorphisms, such as single-nucleotide polymorphisms (SNPs) and a trinucleotide repeat. The association of a (CTG)_n_ repeat polymorphism in the signal sequence of the *CNDP1* gene with DKD was first postulated by Janssen et al., who found that patients with type 2 diabetes carrying the homozygous (CTG)_5_ genotype were less frequently affected by DKD. Hence, this genotype was deemed as a protective factor for developing DKD. The genotype distribution for the (CTG)_n_ repeat is similar in the European and African American populations (Kurashige et al. 2013), yet only European Americans, but not African Americans, homozygous for the (CTG)_5_ allele have a significantly reduced risk for developing diabetic DKD (Freedman et al. 2007b; McDonough et al. 2009). Nonetheless, also in the latter populations, *CNDP1* variants were found to contribute to susceptibility for DKD (McDonough et al. 2009), suggesting that apart from the (CTG)_n_ repeat also SNPs within the *CNDP1* gene, i.e., rs4892247 (McDonough et al. 2009), rs62099905 (Zhang et al. 2020) and rs4891564 (Mota-Zamorano et al. 2021) might influence the course of DKD. The genotype distribution for the (CTG)_n_ repeat strongly differs among ethnicities. While the homozygous (CTG)_5_ genotype is more common in the Caucasian population (homozygous (CTG)_5_ genotype: 34.3%), the homozygous (CTG)_6_ genotype is more prevalent in the South Asian population (Mooyaart et al. 2009) and the most prevalent genotype in the Japanese (Kurashige et al. 2013) and Chinese Han populations (Zhang et al. 2020), with only a small minority carrying the homozygous (CTG)_5_ genotype [Japanese: homozygous (CTG)_6_ vs. homozygous (CTG)_5_ genotypes: 94.7% vs. 0.1%, Chinese Han: homozygous (CTG)_6_ vs. homozygous (CTG)_5_ genotype: 84.5% vs. 0.5%].

The (CTG)_n_ repeat encodes a stretch of leucine amino acids that spans the hydrophobic part of the signal peptide and is required for entering the endoplasmic reticulum and secretory pathway. It is therefore believed that the shorter (CTG)_5_ allelic variant is less efficiently secreted (Riedl et al. 2007), favoring accumulation of carnosine in tissues. Indeed, carriers of the homozygous (CTG)_5_ genotype in general have lower serum carnosinase-1 (CN-1) concentrations and activities (Janssen et al. 2005) which seems to afford protection against renal failure (Albrecht et al. 2017). This may be explained by the relatively higher carnosine, the specific substrate of CN-1, in individuals carried shorter (CTG) allele. There is compelling evidence that carnosine can retard the course of diabetic complications in many ways (Boldyrev et al. 2013; Hipkiss et al. 1994, 2001; Tsai et al. 2010), thereby underscoring the relevance of tissue carnosine concentrations.

In keeping with the forgoing that the genotype frequency of the homozygous (CTG)_5_ genotype is low in the Chinese Han population and that high CN-1 concentration might inflict a risk to develop DKD, we sought to address if other *CNDP1* genotypes are posing a risk for DKD in the Chinese Han population, which factors influence CN-1 concentrations in serum and/or urine, to what extent serum and urinary CN-1 are associated with progression of DKD and finally if CN-1 expression is influenced by diabetes-associated inflammation.

## Materials and methods

### Participants

A total of 651 Chinese Han individuals were recruited in our study, from which 441 diabetic patients were from the Department of Endocrinology, the First Affiliated Hospital of Anhui Medical University from July 2019 to January 2021; 210 subjects without diabetes and renal diseases were taken as controls from Health Management Center in January 2020. This study was approved by the local Ethics Committee (Hefei, China, approval No. PJ2020-15-17). All participants signed written informed consent prior to participating in the study. Out of the total 651 participants, 15 subjects were excluded because of the diagnosis of type 1 diabetes; 7 subjects were excluded because of liver or other kidney diseases; and other 7 subjects were because of urinary tract infections. The remaining subjects (*n* = 622) were subsequently stratified into three groups: 247 T2DM patients without DKD (no-DKD group), 165 T2DM patients with DKD (DKD group), and 210 controls (CON group). The inclusion criteria for T2DM are based on the diagnostic criteria published by WHO, or the patient’s previous medical history. Patients with type 1 diabetes, malignant tumors or autoimmune diseases were excluded. DKD inclusion criteria needed to meet at least one of the following: (1) persistent albuminuria in at least two independent measurements: urinary albumin/creatinine ratio (ACR) not less than 30 mg/g for more than 3 months, (2) eGFR < 60 ml/min/1.73m^2^ for more than 3 months, (3) the pathological diagnosis by renal biopsy. Exclusion criteria of DKD were patients with urinary tract infection, renal disease other than DKD or history of kidney transplantation. Diabetic retinopathy (DR) was screened for each patient by non-mydriatic fundus camera (Canon CR-1 mark II, Japan). Diabetic peripheral neuropathy (DPN) was diagnosed when the conductive speed or conductive amplitude of median nerve/ulnar nerve/radial nerve/peroneal nerve was impaired in the electromyography examination (JB-904BK, Nihon Kohden, Japan). Diabetic macro-vascular disease (DMD) included coronary heart disease, cerebrovascular disease and peripheral artery disease. Peripheral artery disease was explored by Color Doppler ultrasound (Xario 100, Toshiba, Japan) on carotid artery, femoral artery and popliteal artery. The control group consisted of individuals without clinical signs or medical history of renal disease or diabetes. Demographic and clinical data of all participants were collected as shown in Table 1. Serum and urine were collected to measure the concentration of CN-1 and MGO. EDTA blood was used for genotyping. All samples were stored at −20 °C until use.

**Table 1:** The demographic and clinical features of all individuals

	CON	No-DKD	DKD	*p* value
*N*	210	247	165	
Demographic				
Gender (male/female)*	128/82	144/103	111/54	0.181
Age (years)	38.32 ± 0.78	56.26 ± 0.70^a^	59.66 ± 0.87^a^	< 0.001
Clinical				
Weight (kg)	70.09 ± 0.98	67.38 ± 0.75	69.11 ± 0.97	0.072
BMI (kg/m^2^)	24.17 ± 0.25	24.54 ± 0.21	25.02 ± 0.27^a^	0.027
SBP (mmHg)	127.82 ± 1.03	129.59 ± 1.12	139.08 ± 1.76^a,b^	< 0.001
DBP (mmHg)	77.85 ± 0.78	78.65 ± 0.70	82.55 ± 1.08^a,b^	< 0.001
TG (mmol/L)	1.49 ± 0.08	1.91 ± 0.10^a^	1.93 ± 0.10^a^	0.002
TC (mmol/L)	4.57 ± 0.06	4.31 ± 0.07^a^	4.25 ± 0.09^a^	0.011
Diabetes				
Duration of diabetes (years)	–	8.29 ± 0.49	11.35 ± 0.61	< 0.001
FBG (mmol/L)	5.41 ± 0.03	8.01 ± 0.17^a^	8.59 ± 0.26^a^	< 0.001
P2hBG(mmol/L)	–	18.60 ± 0.33	18.76 ± 0.44	0.781
HbA1c (%)	5.48 ± 0.06	9.40 ± 0.15^a^	9.09 ± 0.16^a^	< 0.001
Fasting C peptide (ng/ml)	–	1.01 ± 0.04	1.54 ± 0.14	0.003
Diabetic complications				
Kidney function				
Serum creatinine (μmol/L)	71.22 ± 1.04	61.16 ± 0.94^a^	130.88 ± 11.70^a,b^	< 0.001
Serum BUN (mmol/L)	4.77 ± 0.08	6.12 ± 0.26^a^	8.66 ± 0.44^a,b^	< 0.001
eGFR (ml/min/1.73m^2^)	113.48 ± 1.00	107.43 ± 1.05^a^	67.03 ± 2.77^a,b^	< 0.001
Urinary ACR (mg/g)	–	11.47 ± 0.41	231.59 ± 17.78	< 0.001
24 h-U-PRO(g)	–	0.21 ± 0.01	1.16 ± 0.16	< 0.001
24 h-U-PRO/CRE	–	0.20 ± 0.01	1.25 ± 0.18	< 0.001
DR, *n* (%)*	–	53 (21.54)	50 (30.49)	0.041
DPN, *n* (%)*	–	110 (44.72)	100 (60.98)	< 0.001
DMD, *n* (%)*	–	176 (71.54)	121 (73.33)	0.620
Skin AGEs	–	76.93 ± 0.83	84.17 ± 1.44	< 0.001
CN-1 metabolism				
CN-1 in serum (ng/ml)	0.55 ± 0.03	0.86 ± 0.04^a^	0.74 ± 0.04^a^	< 0.001
Urinary CN-1detection, *n* (%)*	50 (23.81)	106 (42.91)^a^	83 (50.30)^a^	< 0.001
MGO in serum (ng/ml)	2.78 ± 0.17	6.68 ± 0.91^a^	6.02 ± 0.60^a^	< 0.001
Log_10_ (MGO + 1) in serum (ng/ml)	0.52 ± 0.02	0.68 ± 0.02^a^	0.71 ± 0.02^a^	< 0.001

Data are expressed as mean ± SEM, or number

*BMI* body mass index, *SBP* systolic blood pressure, *DBP* diastolic blood pressure, *TG* triglyceride, *TC* total cholesterol, *FBG* fasting blood glucose, *P2hBG* 2 h postprandial blood glucose, *HbA1c* glycosylated hemoglobin, *BUN* blood urea nitrogen, *eGFR* estimated glomerular filtration rate, *ACR* albumin/creatinine ratio, *24 h-U-PRO* 24-h urinary protein, *24 h-U-PRO/CRE* 24-h urinary protein creatinine ratio, *DR* Diabetic retinopathy, *DPN* diabetic peripheral neuropathy, *DMD* diabetic macro-vascular disease, *AGEs* advanced glycation end products, *CN-1* carnosinase-1, *MGO* methylglyoxal

*Chi-square inspection

^a^*p* < 0.05 (post hoc), Compared to CON group

^b^*p* < 0.05 (post hoc), Compared to No-DKD group

### Assessment of serum and urinary CN-1 and serum methylglyoxal concentrations

CN-1 and Methylglyoxal (MGO) were assessed by commercially available Enzyme-linked immunosorbent assays (ELISA) according to the manufacture’s instruction. For CN-1 in serum and urine, the Human Beta-Ala-His Dipeptidase (CNDP1) ELISA Kit (CUSABIO, USA) was used. Serum MGO was measured by the MGO ELISA Kit (LunChangShuo Biotech, China). Each sample was tested in duplicate. Concentrations below the detection limit were defined as negative for CN-1 and MGO.

### Skin AGEs measurement

Skin AGEs were assessed by skin auto-fluorescence at the left volar forearm of the subjects using an Auto-fluorescence Reader (Hefei Institutes of Physical Science, Chinese Academy of Sciences) (Wang et al. 2012). In short, the device used near-ultraviolet light (the center wavelength: 370 nm, the full width at half-maximum: 15 nm) to irradiate skin and stimulate the emission of skin AGEs (range in 420–600 nm). Also, the device emitted a broadband light source in a frequency (wavelength) range of 420–600 nm which illuminated skin so as to measure the tissue illumination absorption and scattering. Emission light, including diffuse reflectance light and fluorescence from the skin was measured by a spectrometer in 300–600 nm range through 3 × 1 fiber bundle. The skin AGEs were finally defined as the ratio of fluorescence intensity and diffuse reflectance light intensity. The range of auto-fluorescence Reader was 0–150. The measurements were performed by well-trained nurses. All measurements were performed on normal skin without visible vessels, scars, lichenification or other skin abnormalities, and at room temperature in a semi-dark environment. For each patient, skin AGEs were assessed in triplicate and expressed as mean auto-fluorescence ± standard deviation (SD).

### *CNDP1* genotyping

Genomic DNA was extracted from whole blood by FlexiGene DNA Kit (QIAGEN, Germany) according to the manufacturer’s instruction. DNA samples were stored at −80 °C until use. The (CTG)_n_ repeat polymorphism was genotyped as previously described (Zhang et al. 2020). Two SNPs within the *CNDP1* gene, i.e., rs4892247 and rs62099905, were analyzed in this study on the basis of previous studies (McDonough et al. 2009; Zhang et al. 2020). The minor allele frequencies (MAF) of these two SNPs were both higher than 0.05 in Chinese populations. SNPs extension primers were consistent with previous studies (Zhang et al. 2020). All SNPs were genotyped using a SNaPshot kit (ABI) in the same way as previously described (Bujalkova et al. 2008).

### Skin inflammation in diabetic mice

A STZ injected C57BL/6 mice model with surgically made skin wound was established to further assess the role of CN-1 in inflammation. After ad libitum feeding for a week, male C57BL/6 mice at week 18 were injected intraperitoneally for 5 consecutive days with Streptozotocin (STZ) (Sigma-Aldrich, St. Louis, MO, USA) at a dose of 50 mg/kg after 12 h fasting each day. STZ was freshly dissolved in sterile citrate buffer (pH 4.5) at doses of 2 mg/ml. The mice with plasma glucose levels higher than 11.1 mmol/L seven days after injection were considered diabetic and used for the experiment. The mice were later randomly divided into two groups: diabetic mice with skin wound (SW group, *n* = 9), diabetic mice without skin wound (non-SW group, *n* = 7). Hereafter, mice at week 20 were anesthetized with intraperitoneal injection of 1% pentobarbital. Two full-thickness 6 mm diameter skin wounds on dorsal skin after shaving were subsequently created by excision. The non-SW group received no treatment. Seven days after the surgery, mice were sacrificed by CO_2_ inhalation. This animal study was approved by the Ethics Committee of Animal Research of Anhui Medical University (Hefei, China, No. PJ2018-05-09). Animal maintenance was conformed to the National Institutes of Health Guide for The Care and Use of Laboratory Animals (NIH Publication No. 8023, revised 1978).

### Histology and immunostaining

Skin wound specimens, kidneys and livers were taken out immediately and fixed with 4% paraformaldehyde, embedded in paraffin, and cut into 5 μm sections until staining. After de-paraffinization and rehydration, skin tissue sections were stained with hematoxylin and eosin (H&E), and then dehydrated through increasing concentrations of ethanol and xylene. Kidney and liver sections were subsequently incubated for 30 min with 0.3% hydrogen peroxide and goat serum to block endogenous peroxidase and unspecific binding respectively. Hereafter the sections were stained overnight for CN-1 by polyclonal antibody (anti-CNDP1 1:100, Proteintech Group, USA), extensively washed and incubated for 30 min at 37 °C with a goat anti-rabbit horseradish peroxidase conjugate (1:100, Tuling Biotech, China). The sections were washed and finally, 3, 3ʹ-di-amino-benzidine (DAB) was used for color development, followed by hematoxylin to stain cell nuclei. Images were captured using a ZEISS microscope and quantified with Image-Pro Plus, version 6.0 (Media Cybernetics, Rockville, MD, USA), software.

### Statistical analysis

Quantitative data were presented as mean ± standard error of mean (SEM). Independent Student’s *T* test and one-way ANOVA were applied for normal distribution data, while Mann–Whitney *U* test and Kruskal–Wallis test were used for non-normal distribution data. Differences for multiple groups were assessed by Bonferroni’s test as a post hoc test. Categorical variables were expressed in numbers or percentages and analyzed using the *χ*^2^ test or Fisher’s exact test. Pearson or Spearman correlation analysis was performed to analyze the correlation between serum CN-1, urinary CN-1, serum MGO, *CNDP1* genotyping and other parameters. Due to the skewness distribution, the serum MGO concentration was logarithmically transformed to log_10_ (serum MGO + 1). In univariate analysis, the variables of a *p* value < 0.25 were selected into the multivariate model. Multivariate linear regression analysis or logistic regression analysis was used to determine the optimal multivariate model for prediction with the dependent variables of serum CN-1, urinary CN-1, log_10_ (serum MGO + 1) and *CNDP1* genotype. The regression coefficients were expressed as 95% confidence intervals (95% CI). All statistical tests were bilateral, *p* < 0.05 was considered statistically significant. The analysis was evaluated using GraphPad Prism 7.0 (GraphPad Software, Inc., La Jolla, California) and SPSS 23.0 Software (SPSS, Inc., Chicago, IL).

## Results

### Demographic and clinical characteristics

A total of 622 subjects were stratified on the basis of clinical criteria into subjects with diabetes and no-DKD (no-DKD, *n* = 247), with diabetes and DKD (DKD, *n* = 165), and no diabetes, no kidneys disease (referred as controls (CON), *n* = 210). Demographic, clinical and serological data are presented in Table 1. No significant difference was observed among the three groups in gender and weight (*p* > 0.05). In the control group, the average age was significantly lower compared to the other two groups (*p* < 0.001), but no difference herein was found between the no-DKD and DKD groups (CON vs. no-DKD vs. DKD: 38.32 ± 0.78 vs. 56.26 ± 0.70 vs. 59.66 ± 0.87 years). Both systolic and diastolic blood pressure were comparable in no-DKD group and CON group, but they were much lower if compared with DKD group (CON vs. no-DKD: *p* > 0.05; no-DKD vs. DKD: *p* < 0.05; CON vs. DKD: *p* < 0.05). Serum triglyceride increased, but serum total cholesterol decreased significantly in no-DKD and DKD group as compared to CON group (CON vs. No-DKD vs. DKD: serum triglyceride: *p* = 0.002; serum total cholesterol: *p* = 0.011).

As expected, patients with diabetes (both no-DKD and DKD group) had higher fasting glucose levels and HbA1c as compared to CON group while patients with DKD had a longer history of diabetes, higher fasting C peptide, higher skin AGEs, higher urinary ACR, higher 24 h urinary protein amount (24 h-UPRO) and higher 24 h urinary protein/creatinine ratio (24 h-UPRO/CRE) as compared to diabetic patients with no kidney disease (DKD vs. no-DKD, *p* < 0.01). Likewise, DR (*p* = 0.041) and DPN (*p* < 0.001) were more prevalent in patients with DKD, while no difference for diabetic macro-vascular disease was found between the two diabetic groups (*p* = 0.620).

Skin AGEs data were not included in CON group as most healthy participants refused to give informed consent to this. Skin AGEs were significantly higher in the DKD group (DKD vs. no-DKD, 84.17 ± 1.44 vs. 76.93 ± 0.83, *p* < 0.001). Unfortunately, skin AGEs were not assessed in controls. Yet, serum MGO were all assessed in each group. The data of serum MGO were not normally distributed. Therefore, log transformation was used. The final results displayed that log-transformed serum MGO accumulated when diabetes and nephropathy developed (CON vs. no-DKD vs. DKD: 2.78 ± 0.17 vs. 6.68 ± 0.91 vs. 6.02 ± 0.60 ng/ml).

Serum CN-1 concentrations in patients with or without DKD were significantly higher than that of the controls and among the patients with diabetes lower in the DKD group (CON vs. no-DKD vs. DKD: 0.55 ± 0.03 vs. 0.86 ± 0.04 vs. 0.74 ± 0.04 ng/ml). Since CN-1 concentration in urine was generally low and even undetectable in most subjects, the percentage of detectable urinary CN-1 in each group was used as a descriptive variable. The prevalence of subjects with detectable CN-1 accounted for 23.81% in the control group and increased gradually in the no-DKD and DKD groups (no-DKD vs. DKD: 42.91% vs. 50.30%, *p* < 0.001).

The compositions of urinary protein as well as albumin-to-creatinine ratio (ACR) and albumin-to-beta-2-microglobulin ratio (Alb/β2-MG) are shown in Table 2. Patients with DKD were further stratified on the basis of ACR into patient with micro-albuminuria (30 mg/g ≤ ACR < 300 mg/g) (*n* = 115) and patients with macro-albuminuria (ACR ≥ 300 mg/g) (*n* = 50). With the exception of NAG and FDP, significant differences were observed in all urinary proteins, being the highest in patients with macro-albuminuria.

**Table 2:** Urinary protein composition of DM patients

	No-DKD	DKD		*p* value
	ACR < 30 mg/g	30 mg/g ≤ ACR < 300 mg/g	ACR ≥ 300 mg/g	
*N*	247	115	50	
Total protein (mg/L)	128.46 ± 5.27	344.84 ± 49.41^a^	884.25 ± 106.76^a,b^	< 0.001
TRF (mg/L)	1.96 ± 0.14	9.95 ± 1.73^a^	31.69 ± 3.92^a,b^	< 0.001
RBP (mg/L)	0.36 ± 0.07	1.67 ± 0.37^a^	4.90 ± 0.85^a,b^	< 0.001
NAG (U/L)	7.27 ± 0.29	8.81 ± 0.58	11.72 ± 1.43^a^	0.002
Alb (g/L)	8.35 ± 1.76	139.44 ± 37.58^a^	464.88 ± 71.61^a,b^	< 0.001
IgG (mg/L)	2.84 ± 0.31	18.95 ± 5.78^a^	74.64 ± 12.73^a,b^	< 0.001
FDP (μg/L)	0.24 ± 0.01	0.20 ± 0.02	0.68 ± 0.24^b^	0.005
Cys-C (mg/L)	0.15 ± 0.01	0.27 ± 0.06	1.00 ± 0.29^a,b^	< 0.001
β2-MG (mg/L)	0.30 ± 0.02	0.56 ± 0.06^a^	1.44 ± 0.22^a,b^	< 0.001
α1-MG (mg/L)	5.89 ± 0.34	10.49 ± 0.73^a^	19.55 ± 1.73^a,b^	< 0.001
ACR (mg/g)	10.49 ± 3.01	149.53 ± 36.45^a^	658.66 ± 99.61^a,b^	< 0.001
Alb/β2-MG (mg/g)	43.79 ± 8.07	355.03 ± 93.46^a^	661.38 ± 188.55^a,b^	< 0.001

Data are expressed as mean ± SEM

*TRF* transferrin, *RBP* retinol-binding protein, *NAG* N-acetyl-glycosaminidase, *Alb* albumin, *IgG* Immunoglobulin G, *FDP* fibrin degradation product, *Cys-C* cystatin C, *β2-MG* beta-2-microglobulin, *α1-MG* alpha-1-macroglobulin, *ACR* albumin-to-creatinine ratio, *Alb/β2-MG* albumin-to-beta-2-microglobulin ratio

^a^*p* < 0.05 (post hoc), Compared to No-DKD group (ACR < 30 mg/g)

^b^*p* < 0.05 (post hoc), Compared to DKD group (30 mg/g ≤ ACR < 300 mg/g)

### *CNDP1* genotyping

The genotyping data of the *CNDP1* (CTG)_n_ repeat polymorphism and two selected SNPs (rs4892247 and rs62099905) of 596 subjects are shown in detail in Table 3. All these three polymorphisms were in Hardy–Weinberg equilibrium (*p* > 0.05). Twenty-six individuals had missing genotyping data, including 19 in the no-DKD and 7 in the DKD groups. The frequencies of different loci, calculated MAF of these loci and the MAF in the 1000 genomes database of the 2 selected SNPs are shown in Table 4. In total, 83.22% of the individuals (496 out of 596) were homozygous for the (CTG)_6_ allele, 15.60% were carriers of one (CTG)_5_ and one (CTG)_6_ allele and only 1.17% had the homozygous (CTG)_5_ genotype. For both SNPs, the homozygous TT genotype was found in the majority of individuals (59.06% and 85.07% for rs4892247 and rs62099905 respectively). Genotype distribution of the (CTG)_n_ polymorphism and SNPs, didn’t differ among the three groups, albeit that the frequency of homozygous (CTG)_5_ genotype slightly but not significantly reduced in the DKD group (CON vs. no-DKD vs. DKD: 1.43% vs. 1.32% vs. 0.63%). Likewise, for rs4892247, the frequencies of homozygous TT genotype increased slightly in both diabetic groups without reaching significance (CON vs. no-DKD vs. DKD: 54.29% vs. 60.09% vs. 63.94%) (Table 3).

**Table 3:** Distribution of polymorphism in *CNDP1* gene

*CNDP1* genotyping	CON	No-DKD	DKD	Total *n* (average %)	*p* value
CTG polymorphism					
(CTG)_5–5_ (%)	3 (1.43%)	3 (1.32%)	1 (0.63%)	7 (1.17%)	> 0.05
(CTG)_5–6_ (%)	33 (15.71%)	37 (16.23%)	23 (14.56%)	93 (15.60%)	
(CTG)_6–6_ (%)	174 (82.86%)	188 (82.45%)	134 (84.81%)	496 (83.22%)	
Total	210 (100%)	228 (100%)	158 (100%)	596 (100%)	
rs4892247					
CC	10 (4.76%)	10 (4.39%)	4 (2.53%)	24 (4.03%)	> 0.05
CT	86 (40.95%)	81 (35.52%)	53 (33.54%)	220 (36.91%)	
TT	114 (54.29%)	137 (60.09%)	101 (63.93%)	352 (59.06%)	
Total	210 (100%)	228 (100%)	158 (100%)	596 (100%)	
rs62099905					
CC	3 (1.43%)	0 (0)	0 (0)	3 (0.50%)	> 0.05
CT	30 (14.29%)	39 (17.11%)	17 (10.76%)	86 (14.43%)	
TT	177 (84.29%)	189 (82.89%)	141 (89.24%)	507 (85.07%)	
Total	210 (100%)	228 (100%)	158 (100%)	596 (100%)	

Data are *n* (%)

**Table 4:** Minor allele frequencies of *CNDP1* SNPs

SNP	N	Minor allele	MAF	MAF in 1000 genomes
rs4892247	596	C	0.225	0.2778
rs62099905	596	C	0.077	0.1755

### Factors influencing serum and urinary CN-1 concentrations

**Fig. 1:**
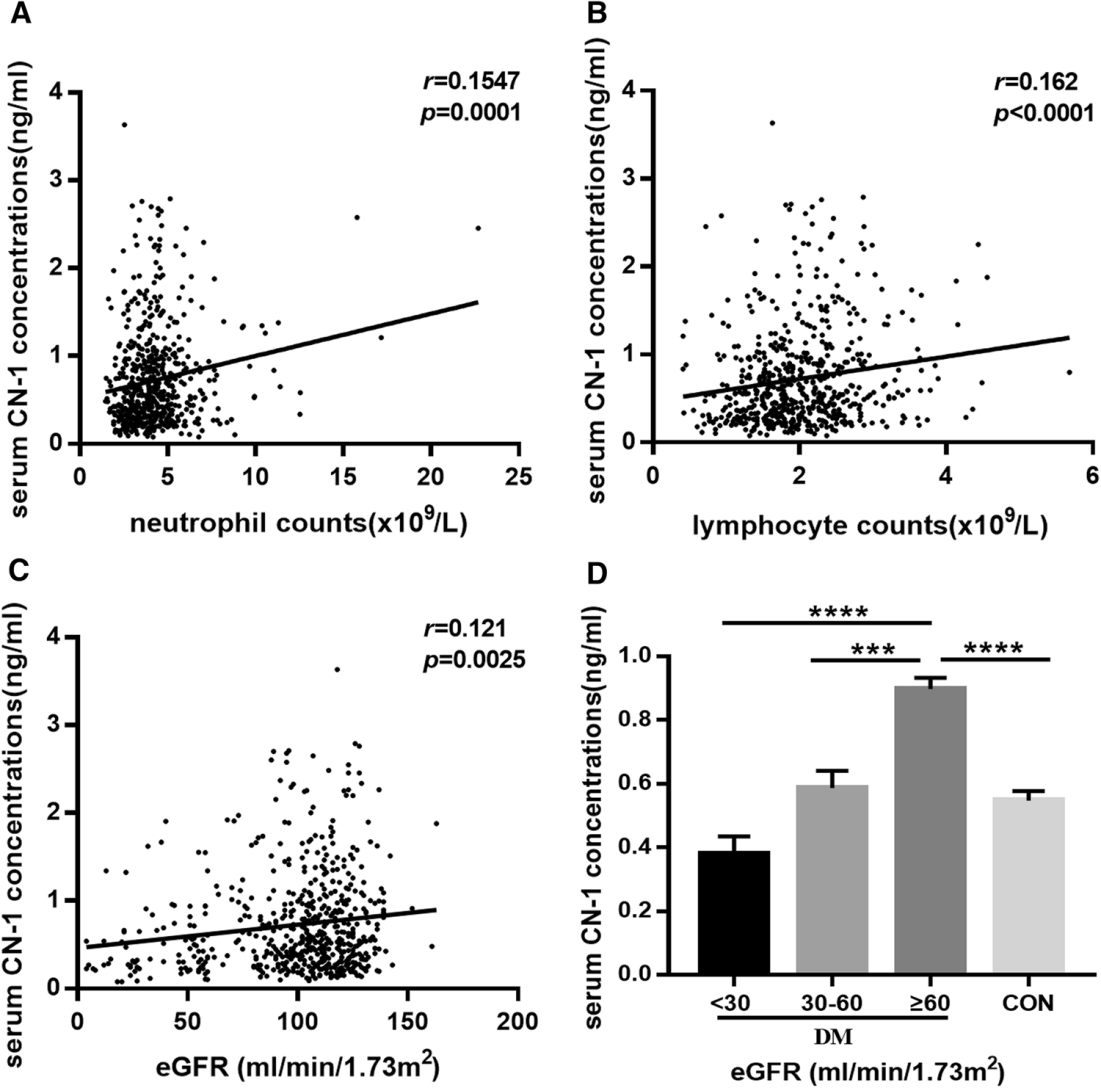
Serum CN-1 concentrations was associated with kidney function and inflammation. Correlations between serum CN-1 concentrations with circulating neutrophil counts (**a**), lymphocyte counts (**b**), eGFR (**c**) in all subjects. Serum CN-1 also increased as eGFR increased in diabetic patients (**d**), while the control group had significantly lower serum CN-1 than diabetic patients with good renal impairment (eGFR ≥ 60 ml/min/1.73m^2^, **d**). Data represent means ± SEM. ***p < 0.001 (post hoc); ****p < 0.0001 (post hoc)

To assess genetic and clinical factors that correlate with serum CN-1 concentrations, we performed Spearman correlation analysis between serum CN-1 and genetic and clinical parameters. Due to the small sample size for the homozygous (CTG)_5_ genotype (*n* = 7) and for the homozygous rs62099905 CC (*n* = 3) and rs4892247 CC genotypes (*n* = 24), they were excluded from the analysis. Variables with a *p* value less than 0.25 were subsequently selected for multivariate linear regression analysis. As shown in Table 5, eGFR, circulating neutrophil and lymphocyte counts were independent determinants of serum CN-1 concentrations. As shown in Fig. 1, there was a significant positive correlation between serum CN-1 concentrations and eGFR (*r* = 0.121, *p* = 0.0025), and neutrophil (*r* = 0.1602, *p* < 0.0001) and lymphocyte counts (*r* = 0.1547, *p* = 0.0001) (Fig. 1a–c). No correlation between serum CN-1 and MGO was found.

**Table 5:** Summary of uni- and multi-variate analysis of serum CN-1 in all subjects

Variables	Univariate analysis		Multivariate analysis	
	R	*p* value	B (95% CI)	*p* value
Neutrophil counts (× 10^9^/L)	0.155	< 0.001	0.062 (0.037, 0.088)	< 0.001
Lymphocyte counts (× 10^9^/L)	0.162	< 0.001	0.109 (0.038, 0.180)	0.003
eGFR (ml/min/1.73m^2^)	0.121	0.003	0.003 (0.001, 0.005)	0.001

To further explore the relationship between serum CN-1 concentration and renal function, all patients with diabetes were stratified according to eGFR. Patients with good renal function (eGFR ≥ 60 ml/min/1.73m^2^, *n* = 320) had significantly higher serum CN-1 concentration than patients with moderately decreased renal function (eGFR ranging from 30 to 60 ml/min/1.73m^2^, *n* = 61) (*p* = 0.0007) and patients with severely impaired renal function (eGFR < 30 ml/min/1.73m^2^, *n* = 31) (*p* < 0.0001). Compared with the control subjects with no diabetes, patients with good renal function (eGFR ≥ 60 ml/min/1.73m^2^) had significantly higher serum CN-1 concentrations (*p* < 0.0001) (Fig. 1d).

**Fig. 2:**
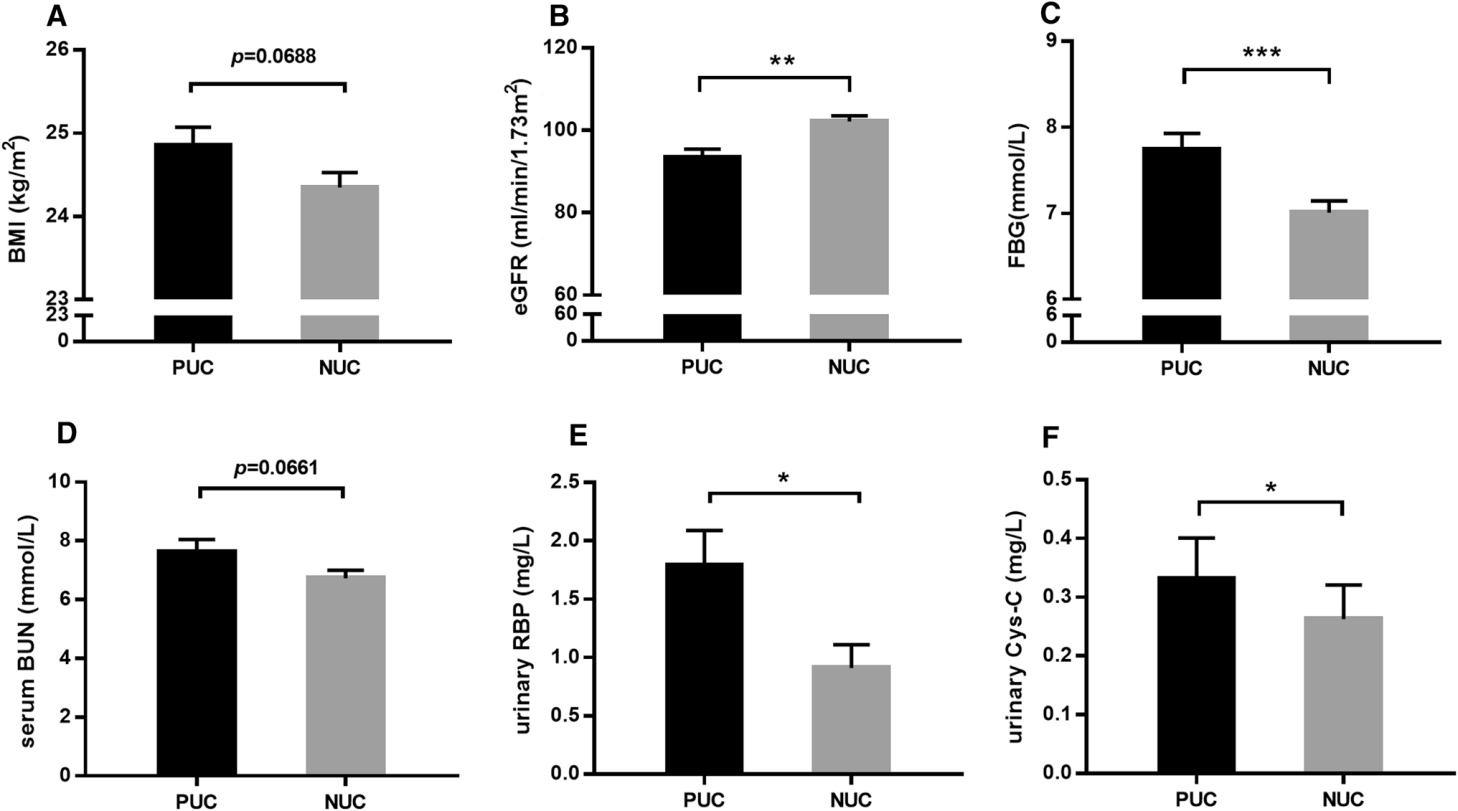
Relationship between PUC and NUC groups in terms of different clinical indicators. PUC group displayed higher BMI levels (**a**), higher FBG levels (**c**) but lower eGFR levels (**b**) as compared to NUC group in all subjects. In T2DM patients, PUC group showed higher serum BUN (**d**), urinary RBP (**e**) and urinary Cys-C (**f**) levels. Data represent means ± SEM. *p < 0.05; **p < 0.01; ***p < 0.001

According to the presence or absence of urinary CN-1, subjects were divided into two groups: positive urinary CN-1 (PUC, *n* = 239) and negative urinary CN-1 (NUC, *n* = 382) groups. Spearman correlation analysis and binary logistic regression analysis were carried out between urinary CN-1 and all other parameters. In the multivariate model, urinary CN-1 was associated with BMI, eGFR and FBG (Table 6). The PUC group displayed higher BMI, higher FBG but lower eGFR levels (Fig. 2a–c, PUC vs. NUC: BMI (kg/m^2^): 24.85 ± 0.22 vs. 24.35 ± 0.18; FBG (mmol/L): 7.74 ± 0.19 vs. 7.00 ± 0.14; eGFR (ml/min/1.73m^2^): 93.29 ± 2.14 vs. 102.13 ± 1.38). For the association of urinary proteins and urinary CN-1, the control group was excluded since protein composition in urine was only assessed in the two diabetic groups. Univariate and multivariate analyses disclosed an association between urinary CN-1, urinary RBP and urinary Cys-C. When all serological data were included, the relevance of urinary CN-1 and serum BUN was also found in exclusive diabetic patients (Table 7). The PUC group showed higher serum BUN and higher urinary RBP and Cys-C levels as compared to NUC group in all diabetic patients (Fig. 2d–f, PUC (*n* = 189) vs. NUC (*n* = 222): serum BUN (mmol/L): 7.63 ± 0.42 vs. 6.73 ± 0.27; urinary RBP (mg/L): 1.79 ± 0.30 vs. 0.91 ± 0.20; urinary Cys-C (mg/L): 0.33 ± 0.07 vs. 0.26 ± 0.06).

**Table 6:** Summary of uni- and multi-variate analysis of Urinary CN-1 in all subjects

Variables	Univariate analysis		Multivariate analysis		
	R	*p* value	B	OR (95% CI)	*p* value
BMI (kg/m^2^)	0.074	0.069	0.054	1.055 (1.002, 1.112)	0.042
eGFR (ml/min/1.73m^2^)	−0.115	0.004	−0.010	0.990 (0.985, 0.996)	0.001
FBG (mmol/L)	0.138	0.001	0.082	1.085 (1.019, 1.156)	0.011

**Table 7:** Summary of uni- and multi-variate analysis of Urinary CN-1 in DM patients

Variables	Univariate analysis		Multivariate analysis		
	R	*p* value	B	OR (95% CI)	*p* value
Serum BUN (mmol/L)	0.060	0.227	0.169	1.184 (1.055, 1.329)	0.004
Urinary RBP (mg/L)	0.107	0.065	0.237	1.267 (1.060, 1.516)	0.010
Urinary Cys-C (mg/L)	0.118	0.044	−0.660	0.517 (0.287, 0.931)	0.028

### Tissue CN-1 expression is increased in diabetic mice carrying surgical skin wounds

**Fig. 3:**
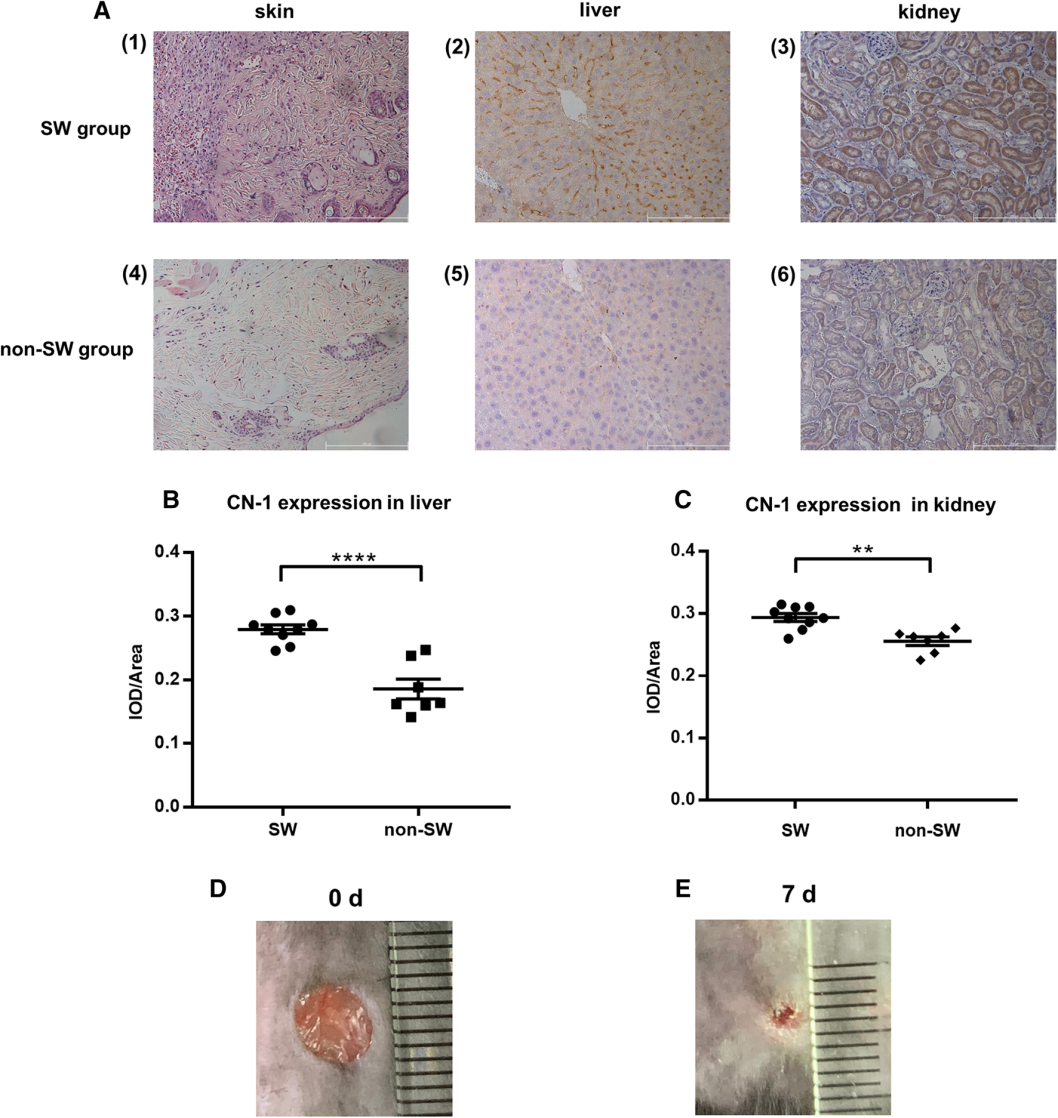
Expression of CN-1 in liver and kidney tissues was increased in diabetic mice with skin wound. HE staining of mice skins and immunohistochemical (IHC) staining of CN-1 in liver and kidney tissues (**a**). Representative microscopic images are shown (magnification, 200 ×). Immunohistochemical results expressed as integrated optical density/total area (IOD/Area) (**b, c**). Skin wound area changes (**d, e**) in diabetic mice. Data represent means ± SEM. **p < 0.01; ****p < 0.0001

Since it was found that serum CN-1 concentrations correlate with increasing circulating neutrophils and lymphocytes, we sought to elucidate this in vivo by addressing if CN-1 expression would increase in diabetic mice with or without inflammatory skin lesions. To this end, we established a diabetic mice model with dorsal wound cut (Fig. 3d) as described in materials and methods part. CN-1 is not expressed in serum of rodents because it lacks a signal peptide, we therefore assessed CN-1 expression in liver and kidney. Mice were sacrificed one week after inflicting wounds, a time at which signs of wound healing appeared (Fig. 3e). Diabetic mice carrying surgical wounds (SW) did not differ in FBG from diabetic mice without surgical wounds (no-SW) at the time of sacrifice (no-SW vs. SW: FBG (mmol/L): 27.51 ± 1.19 vs. 27.88 ± 1.25). HE staining demonstrated a large number of inflammatory cells accumulation around the wounds (Fig. 3a). CN-1 expressions in liver and kidney tissues were examined by immuno-histochemistry. As shown in Fig. 3a-3 and 3a-6, CN-1 was mainly expressed in proximal—and distal tubular epithelial cells. Moreover, CN-1 expressions in liver and kidney remarkably increased when diabetic mice had skin wound (Fig. 3a-2, a-3, a-5, a-6) as demonstrated by morphometric analysis (Fig. 3b, c).

## Discussion

Protection against DKD afforded by variants in the *CNDP1* gene has been reported in many studies across different ethnicities, albeit not always related to the *CNDP1* (CTG)_n_ polymorphism (Chakkera et al. 2011; Guo et al. 2016; Kurashige et al. 2013; Tziastoudi et al. 2020; Yadav et al. 2016; Yahya et al. 2019). The (CTG)_n_ polymorphism is situated in the signal sequence of *CNDP1* and likely affects secretion of CN-1. We have previously shown that this allele has a low frequency in the Chinese Han population (Zhang et al. 2020), which makes assessment of protection for this allele in this population cumbersome. Because the Chinese Han population has a high incidence of DKD and a low frequency of the homozygous (CTG)_5_ genotype, this study therefore sought to address if other *CNDP1* genotypes are posing a risk for DKD in the Chinese Han population. Moreover, since high serum CN-1 concentrations seem to be a risk factor for DKD, we also assessed which factors influence CN-1 concentrations in serum and/or urine, to what extent serum and urinary CN-1 are associated with progression of DKD and finally if CN-1 expression is influenced by diabetes-associated inflammation.

In line with previous reports on genotype distribution in Asians (Yahya et al. 2019; Zhang et al. 2020), our present data confirmed that the homozygous (CTG)_5_ genotype is indeed rare (1.17%) in the Chinese Han population. No statistical significance for association of the (CTG)_n_ polymorphism or the studied SNPs with DKD was found, yet not all SNPs in *CNDP1* have been systematically studied. We have chosen to focus on the rs4892247 and rs62099905 SNPs because of the relatively high MAF in the Chinese population of the former and because previous publication suggested an association of this SNP with DKD in Chinese (Guo et al. 2016). The latter SNP showed an association with FBG levels in healthy Han Chinese (Zhang et al. 2020). An association with serum CN-1 concentrations or with the presence of CN-1 in urine and *CNDP1* genotype was also not found. Despite a lack of association between *CNDP1* variants and DKD or serum CN-1 concentrations, our study did reveal a role of the latter in the progression of DKD. Similar to our previous studies (Zhang et al. 2019; Zhou et al. 2021), serum CN-1 concentrations in T2DM patients with poor or intermediate renal function (eGFR < 60 ml/min/1.73m^2^) were significantly lower as compared to those with good renal function (eGFR ≥ 60 ml/min/1.73m^2^), but the latter patients had higher serum CN-1 concentrations compared to healthy controls (Fig. 1d). This fits with the assumption that high serum CN-1 concentration confers a risk to develop DKD (Zhou et al. 2021). As DKD progresses serum CN-1 concentrations fall, possibly as a consequence of urinary excretion in combination with protein energy wasting (Rodriguez-Niño et al. 2019a, b). The presence of urinary CN-1 in subjects with poor eGFR (Fig. 2b) further supports the forgoing hypothesis. Hence, our current results in the Chinese Han population corroborate and extent previous associations of carnosinasuria with indicators of glomerular function deterioration, e.g., eGFR and serum urea nitrogen (Fig. 2b, d). Our study also showed that carnosinasuria increased in parallel with tubular injury markers, i.e., urinary RBP and Cys-C (Fig. 2e, f), which are reported to predict progression to end-stage renal disease in type 2 diabetes mellitus with advanced nephropathy (Satirapoj et al. 2019). In keeping with our current findings and previous studies that serum CN-1 correlates with renal tubular kidney injury molecule (KIM-1) staining of biopsies from patients with DKD, serum and urinary CN-1 might be a surrogate marker for both glomerular and tubular injury that to some extent may predict the course of DKD.

An interesting finding of our current study was the observation that serum CN-1 concentrations correlated with circulating neutrophil and lymphocyte counts. Although the number of neutrophils and lymphocytes were not in a pathological range, they might be indicative of a low-grade sub-clinical inflammation which often accompanies patients with diabetes. This prompted us to establish a model of acute skin inflammation, caused by surgical wounds, in diabetic mice to assess if this would influence CN-1 expression. CN-1 expression in liver and kidney was significantly increased in diabetic mice carrying surgical wounds, suggesting that a low-grade inflammatory condition indeed may affect CN-1 expression at least in mice. Since carnosine may alleviate inflammation as previously reported (Fleisher-Berkovich et al. 2009; Liu et al. 2020), a correlation between pseudo-inflammatory variables and the carnosine degrading enzyme in the Chinese Han population was unexpected. A correlation between low-grade inflammation and serum CN-1 expression has been recently suggested in obese non-diabetic individual (MaynerisPerxachs et al. 2021).

In conclusion, our study for the first time found an association between circulating neutrophils/lymphocyte counts and CN-1 levels in diabetic patients. Although the underlying mechanism to explain the correlation is not fully clear, it seems that inflammatory processes might affect serum CN-1 as also suggested by others (MaynerisPerxachs et al. 2021). In keeping with current knowledge of CN-1 in the context of diabetes and low-grade inflammation, further studies are warranted to reinforce the role of CN-1 herein.

## Supplementary Information

Below is the link to the electronic supplementary material.

Supplementary file1 (XLSX 243 KB)

## Data Availability

The clinical data used to support the conclusions of this study are restricted by the local ethics committee to protect the privacy of subjects. Data are available from Dr. Shiqi Zhang (zhangshiqi@ahmu.edu.cn) for researchers who meet the criteria for access to confidential data.
